# MRI Stereoscope: A Miniature Stereoscope for Human Neuroimaging

**DOI:** 10.1523/ENEURO.0382-21.2021

**Published:** 2022-02-14

**Authors:** I. Betina Ip, Ivan Alvarez, Mike Tacon, Andrew J. Parker, Holly Bridge

**Affiliations:** 1 Wellcome Centre for Integrative Neuroimaging, FMRIB Building, Nuffield Department of Clinical Neurosciences, University of Oxford, John Radcliffe Hospital, Oxford OX3 9DU, United Kingdom; 2 Helen Wills Neuroscience Institute, University of California Berkeley, Berkeley 94720, CA; 3 Denys Wilkinson Building, Department of Physics, University of Oxford, Oxford OX1 3RH, United Kingdom; 4 Department of Physiology, Anatomy and Genetics, University of Oxford, Oxford OX1 3PT, United Kingdom; 5 Institut für Biologie, Otto-Von-Guericke Universität, Magdeburg 39120, Germany

**Keywords:** binocular disparity, dichoptic presentation, mirror stereoscope, MRI scanner, open-source, visual display

## Abstract

Stereoscopic vision enables the perception of depth. To study the brain mechanisms behind stereoscopic vision using noninvasive brain imaging (magnetic resonance brain imaging; MRI), scientists need to reproduce the independent views of the left and right eyes in the brain scanner using “dichoptic” displays. However, high-quality dichoptic displays are technically challenging and costly to implement in the MRI scanner. The novel miniature stereoscope system (“MRI stereoscope”) is an affordable and open-source tool that displays high-quality dichoptic images inside the MRI scanner. The MRI stereoscope takes advantage of commonly used display equipment, the MRI head coil, and a display screen. To validate the MRI stereoscope, binocular disparity stimuli were presented in a 3T MRI scanner while neural activation was recorded using functional MRI in six human participants. The comparison of large binocular disparities compared with disparities close to zero evoked strong responses across dorsal and ventral extra-striate visual cortex. In contrast, binocularly anti-correlated stimuli, which are not perceived in depth, did not evoke comparable activation. These results are the proof-of-concept that the MRI stereoscope can deliver dichoptic images that produce the perception of stereoscopic depth during acquisition of MR responses. Application of the MRI stereoscope to neuroscience can help to address important questions in perception and consciousness.

## Visual Abstract

**Figure:**
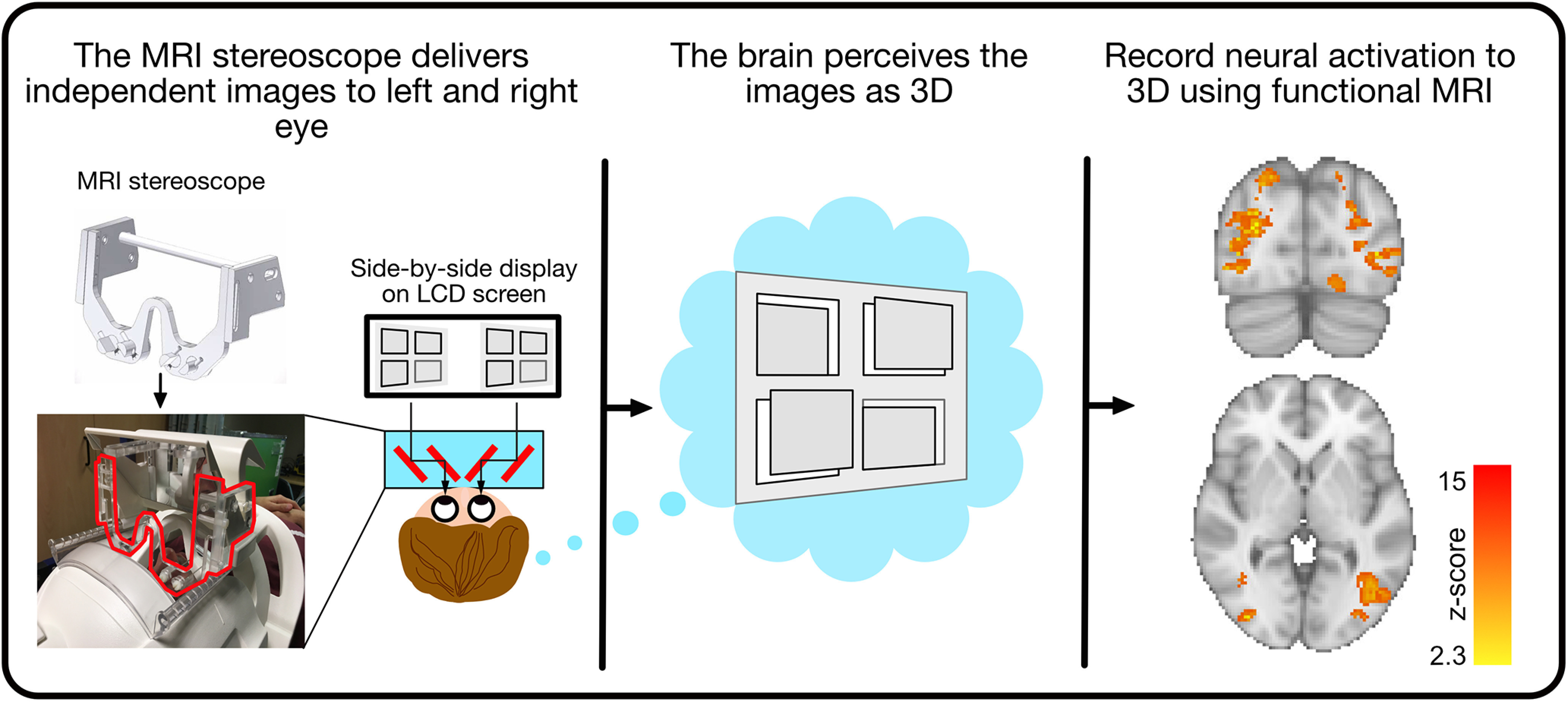

## Significance Statement

Independent stimulation of the left and right eye is a prerequisite to studying perception and consciousness through the binocular visual system. Yet, such “dichoptic” displays are not widely used in neuroimaging because of the technical challenges of delivering high-quality dichoptic displays in the magnetic resonance brain imaging (MRI) scanner. We present a demonstration of a novel, miniature MRI stereoscope, produced in a mechanical workshop using commonly available materials that overcomes these challenges. Our device can accelerate research into the biological basis of binocular vision by providing a low-cost, high-quality device to display dichoptic images for neuroscientific research.

## Introduction

### Binocular vision

The two forward-facing eyes of humans allow us to perceive the world in solid depth, with the small differences between the images arriving at the left and right eyes supporting the perception of the third dimension. Binocular stereopsis makes our interaction with the world precise and vivid. For example, the precision of binocular stereopsis enhances clinical medicine, from visual detection of abnormalities in radiology images, to improving hand-eye-coordination in the surgical theater (Held and Hui, 2011). For neuroscience, the binocular visual system is an ideal model system to investigate neuronal computation in the neocortex, since the information from the two eyes is combined for the first time in the primary visual cortex. Full experimental control over binocular input is critical for research into binocular depth perception, as well as studies of consciousness and binocular processing using binocular rivalry (Carmel et al., 2010; Law et al., 2013) and continuous flash-suppression (Tsuchiya and Koch 2005; Yuval-Greenberg and Heeger, 2013). While much of the investigation of the binocular visual system has been performed in animals using neurophysiology, brain imaging has allowed researchers to investigate neural processing across the visual hierarchy in the human brain.

One of the most widely used methods is noninvasive magnetic resonance brain imaging (MRI), for which separate and independent visual inputs to each eye (dichoptic) inside the scanner room are necessary to study the binocular visual system. Displaying visual images within the MRI environment is technically challenging: hardware inside the scanner room needs to (1) be nonferromagnetic to ensure the safety of participants and radiographers; (2) cause no distortions of the magnetic fields to avoid affecting data quality; (3) remain fully functional within an extremely high magnetic field environment; and (4) must also be compact enough to fit within the severely restricted space around the participant’s eyes and the MRI head coil that is used to record signals from the brain (Roby et al., 2000). Because the human binocular disparity system is sensitive to extremely small differences in the images from left and right eye, it is of key importance to optimize for independence and precision of stereoscopic images in dichoptic displays.

### Existing approaches for dichoptic display in the MRI scanner

All dichoptic displays need to provide precise and independent control over the images seen by each eye, such that one eye can only see the image presented to that eye alone. The manner in which various approaches achieve this separation differ and can be broadly categorized into “overlapping” and “nonoverlapping” stereo-displays (Banks et al., 2016). The main approaches used for MRI experiments are mentioned below. In overlapping stereo-displays, images for the left and right eye occupy the same pixel positions on the display screen, but the information presented to each eye is separated into segregated channels via spectral banding, polarized light or through flicker at different temporal frequencies. Although overlapping stereo-displays take advantage of the entire display screen size and afford excellent visual field coverage, there is an important trade-off with image cross talk (Woods, 2012). Cross talk is the appearance of the image intended for viewing by one eye leaking to the other eye as a “ghost” image. Cross talk not only decreases image quality and is non-naturalistic, but can also impair binocular depth perception (Tsirlin et al., 2011), as well as affect viewer comfort during viewing of binocular displays (Kooi and Toet, 2004). In anaglyph stereo that uses color to separate monocular images, cross talk is created by insufficient separation of the color channels.

The problem of cross talk is eliminated in nonoverlapping stereo-displays, in which the left and right eye’s images are separated by spatial location. Nonoverlapping stereo displays include mirror stereoscopes and prism goggles. However, spatial separation comes at the expense of visual field coverage, as each eye’s image occupies one half of the visual display. Because of the complete spatial separation, these methods need to ensure that proper binocular alignment is achieved before the experiment commences. The above-mentioned dichoptic display approaches take advantage of an existing MRI compatible front-or back-projection system. Further pros/cons for each of these display approaches are listed in Table 1 and are discussed in greater detail in previously published reviews (Carmel et al., 2010; Law et al., 2013; Banks et al., 2016). The cost of a dichoptic display system is another important consideration (Law et al., 2013) and has led to a number of alternative solutions using polarized light (Thompson et al., 2008) and prism goggles (Schurger, 2009). At the upper end of the price range are commercially available head-mounted displays (HMDs) that use small monitors to display images independently to each eye. HMDs overcome both the problems of cross talk and reduction in visual field, but are costly, and are not always easy to align well enough to simulate natural viewing conditions. Because of MR safety requirements, HMDs rely on fiber optic technology to show externally presented images inside the scanner (Cornelissen et al., 1997). Fiber optic cables are made of thin, transparent strands of plastic or glass that do not cause radio frequency disturbances. However, fiber optic cables are delicate and prone to damage, which leads to image artefacts that are difficult to repair, such as light spray and dead pixels. The ongoing requirement for external technical support is another caveat for HMDs.

**Table 1:** Main advantages and disadvantages of dichoptic display approaches in the MRI scanner

	Type	Separation	Main Pros	Main Cons	Cost
1	Anaglyph	Spectral: colored filters are used to separate input to each eye: for example, the red filter passes red image and the green filter passes the green image	• Affordable and easy to use • Independent of head position • Full size image	• Images can only be grayscale, not suitable for images in color • Non-naturalistic • Prone to cross talk • Limited in luminance range	$1.00
2	Passive polarizing glasses	Polarized light: orthogonal polarization of light going to left and right eyes	• Full color display • Full size image • Can be used concurrently with eye tracking	• May affect color display (circular polarization) • Dependent on optimal head position (linear polarization) • Flicker artefact caused by temporal asynchrony • Prone to cross-talk	$7000 (Thompson et al., 2008)
3	Mirror stereoscope	Spatial: angled mirrors are used to display one half of the screen to each eye	• Independent display to each eye • Zero cross talk • Full color • Duplicates screen properties, no distortion of visual display properties • Precise	• Visual field is halved • Needs stationary head position • Needs calibration for each participant • Difficult to use with concurrent eye tracking	$4260
4	Prism lenses	Spatial: the optical path is diverted using prisms so each eye fixates one half of the screen	• Affordable • Independent display to each eye • Zero cross talk • Full color	• Distortion at periphery of visual field^12^ • Limited field of view • Needs stationary head position • Cannot be used for very large disparities • Difficult with concurrent eye-tracking	$300 (Schurger, 2009)
5	Head-mounted display	Spatial: small display screens are presented in front of each eye in a goggle system	• Full color • No distortion of visual display properties • Full display size • Integrated eye-tracking	• High cost • Requires continuous external support and technical assistance	$100,000

Rows 1 and 2 are “overlapping” stereo-displays. Rows 3–5 are “nonoverlapping” stereo-displays that work using spatial separation. Cost estimates are in United States dollar (USD) and approximate. $ = USD.

Real objects are defined by multiple binocular and monocular depth cues. This natural relationship is broken by any kind of dichoptic display, which artificially recreates specific depth cues on a flat screen. The displayed dichoptic images conflict with static depth cues from the flat screen, because accommodation and blur of the images, as well as vergence eye movements during scene exploration, are related to a display screen rather than the 3D image. The discrepancy causes well documented instances of visual discomfort and fatigue (Lambooij et al., 2009). Certain depth cues remain difficult to reproduce by dichoptic displays in the MRI scanner, including depth from focus, accommodation cues that depend on the viewing distance to objects, and retinal blur, which relies on the gradient of blur of real objects in space. Motion parallax, a monocular depth cue that is created by head motion or object motion, can only be visually simulated as a stable head position is necessary for MRI scanning. These limitations, common to all dichoptic displays in the MRI scanner, can be more readily studied and overcome in the psychophysical laboratory (Hoffman et al., 2008).

### Mirror stereoscopes for the MRI

Mirror stereoscopes, invented by Charles Wheatstone (Wheatstone, 1838; Wade, 2012), use mirrors to achieve physical separation of the images to the two eyes for dichoptic presentation. They provide precise and accurate dichoptic displays with flawless reproduction of the image in the psychophysical laboratory, but are thought to be incompatible with the MRI-environment because stereoscopes usually contain ferromagnetic materials and are relatively large in size (Carmel et al., 2010). While previous fMRI studies have used stereoscopes (Polonsky et al., 2000; Brouwer et al., 2005; Lee et al., 2005, 2007; Sterzer et al., 2008; Moradi and Heeger 2009); the methodological descriptions did not contain sufficient detail to reproduce the instruments. We are the first to provide a detailed description of a miniature MRI compatible stereoscope, complete with *in vivo* validation of the device.

### Our system

We designed a novel MRI stereoscope system (RRID:SCR_021655) by attaching two pairs of small, angled mirrors via a custom-made mirror holder, on a 64-channel head coil of a 3T Siemens Prisma scanner. We validated the capacity of the MRI stereoscope for dichoptic display using dynamic random dot stimuli (RDS) defined by binocular disparity. In RDS, the perception of depth arises solely by fusing the left and right eye’s image and extracting the small differences as binocular disparity depth cues. If an RDS with non-zero binocular disparity is viewed with one eye only, it will appear as a flat field of dots. The main limitations of the instrument are the reduced field-of-view, effectively halving a display screen, and the lack of support for concurrent eye-tracking, because of mechanical limitations in capturing images of the eyes through the stereoscope with a long-distance infrared (IR) eye tracker.

## Materials and Methods

### Description of the MRI stereoscope

**Figure 1.**
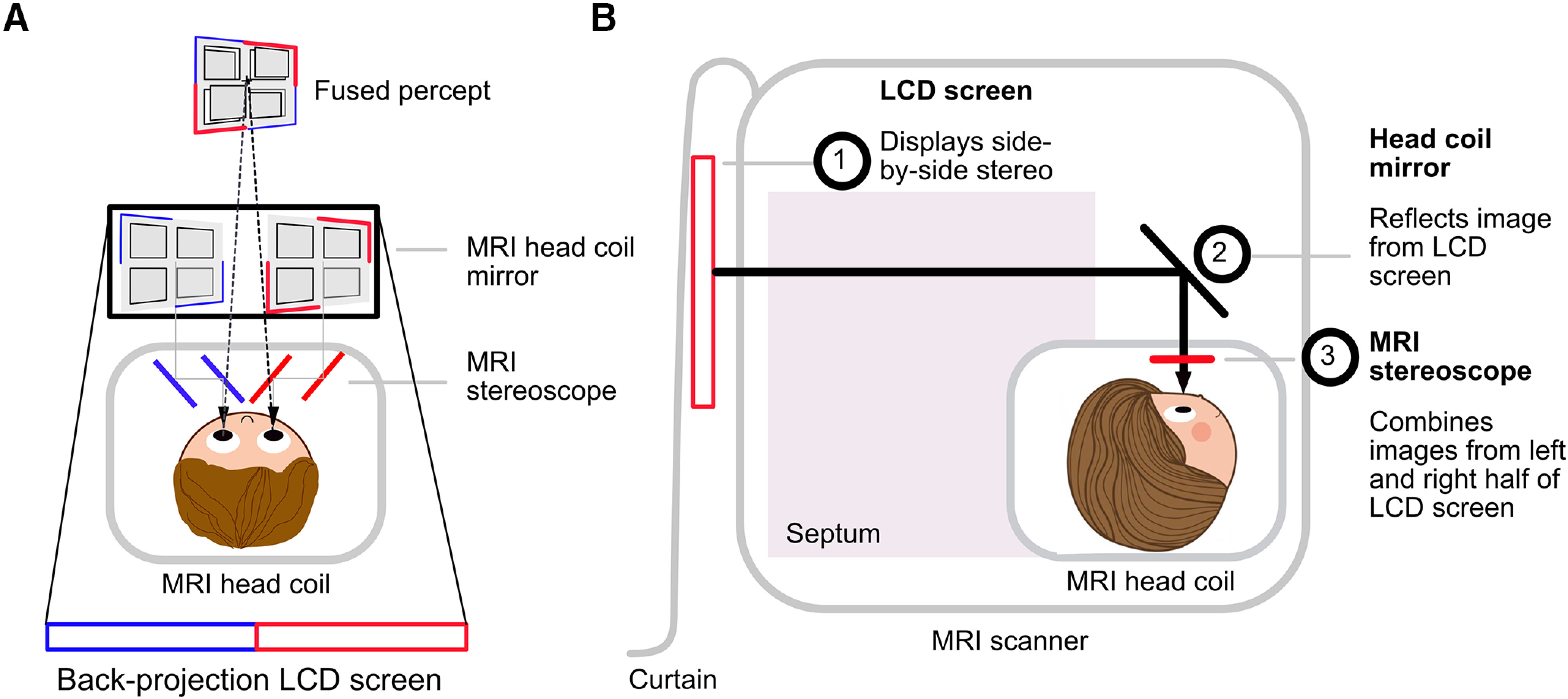
Concept diagrams of MRI stereoscope system. ***A***, Bird’s eye view of the MRI stereoscope. When viewed from behind the liquid crystal display (LCD) screen, the left half of the LCD screen (red) is seen by the left eye, and the right half (blue) by the right eye. The head coil is indicated with the gray box. By viewing the images through the stereoscope, the participant fuses the left and right half images to perceive a unitary image. ***B***, Schematic diagram for whole set-up installed in the scanner as seen from the right side of the participant, including LCD screen (1), head coil mirror (2), and MRI stereoscope (3), as well as septum and a blackout curtain.

The principle of the MRI stereoscope is shown in Figure 1*A*. Images are presented side-by-side on the LCD screen, and viewed through small, angled mirrors positioned close to the eyes. Viewed through the mirrors, the left half of the screen is seen by the left eye and the right half of the screen by the right eye. The size of the LCD screen active area was 698 × 393 mm (resolution 1920 × 1200, aspect ratio = 8:5, refresh rate = 60 Hz) and the viewing distance for the participant was 127 cm. The binocularly-fused display thus subtended 10.23° × 12.88° of visual angle. The size of the projection bore (60 cm radius) set a limit on the size of the visible screen, which in turn set a limit on the size of stimuli that could be displayed in a side-by-side stereo mode. We used “stereo mode” from MATLAB (v8.0, MathWorks Inc.) Psychtoolbox-3 (Brainard 1997; Pelli 1997; v3.0, http://psychtoolbox.org) to display visual stimuli in side-by-side stereo. In our validation experiment, we used dynamic random-dot-stereograms to measure responses to binocular disparity in the MRI scanner.

The entire stereoscope system is schematically presented in Figure 1*B*. It was installed on a 3T Siemens Prisma scanner (Siemens Healthcare) using a Siemens 64-channel head coil with a modified 64-channel Head/Neck coil rear-view mirror holder, and all components were inside the scanner room. An MRI compatible LCD screen (BOLDscreen 32, Cambridge Research Systems) was positioned as close as possible to the scanner at the back of the bore and a blackout curtain was draped over the back of the LCD screen. The curtain was made of black cotton cloth that was fixed to the back of the scanner using adhesive tape. The purpose of the curtain was to prevent the images surrounding the screen from being seen, which would lead to double vision. A septum made of rigid plastic ran from the LCD screen to the top of the head coil, dividing the visual field in two and preventing reflections from one side of the bore affecting the other. Small brackets running along the length of the bore were added to hold the septum in place. The standard rear-silvered mirror that presented images at a 45° angle was replaced by a custom-made front-silvered mirror to prevent ghosting of the image through two reflective surfaces. The MRI stereoscope was mounted between the head coil mirror and the 64-channel head coil. In the final design stage, black paper baffles were constructed around the mirror images to prevent images from other regions from interfering with binocular viewing.

### Construction of the MRI stereoscope

**Figure 2.**
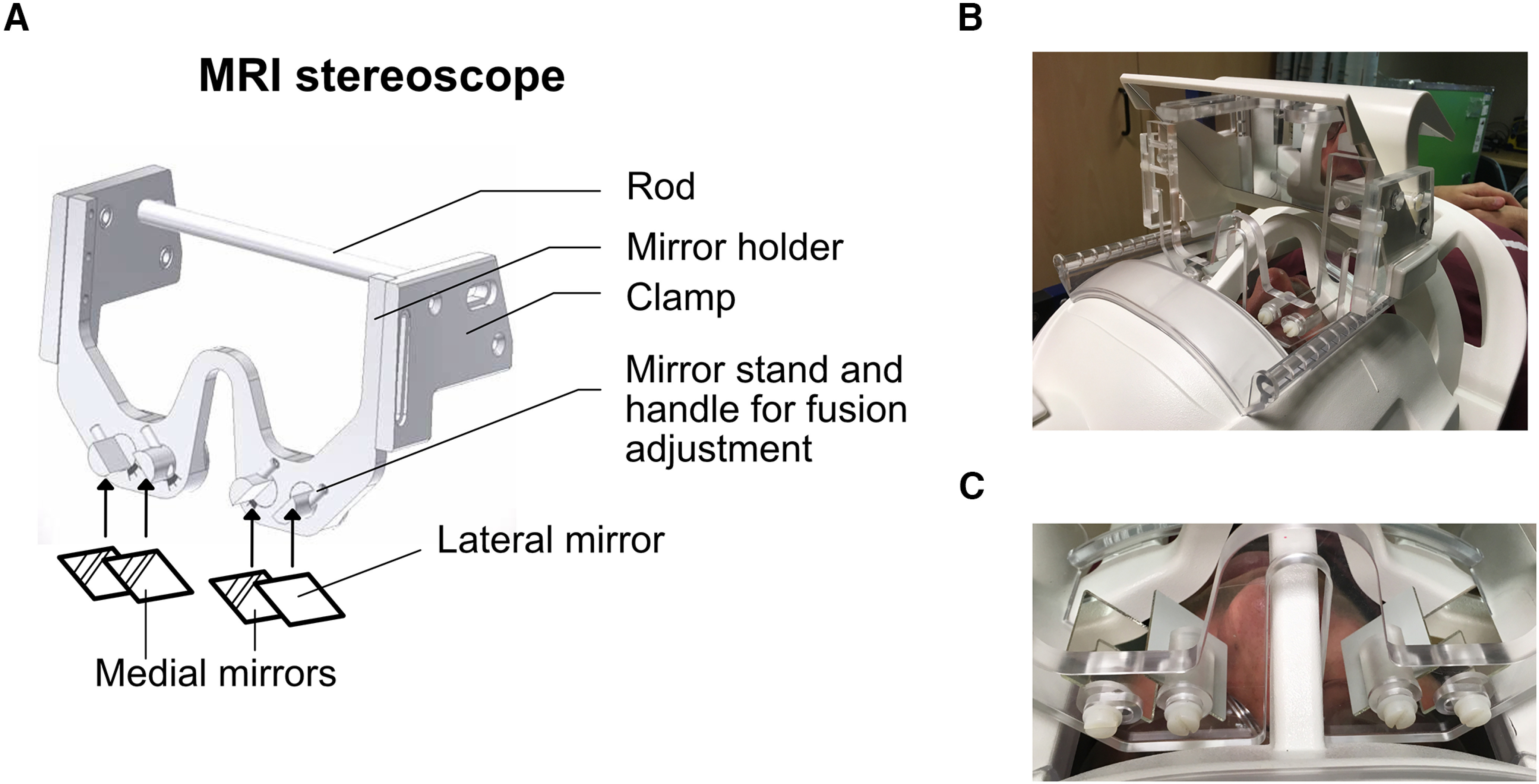
Technical drawing of MRI stereoscope and photograph of prototype. ***A***, Technical drawing of the MRI stereoscope with mirrors drawn as diagrams. A rod is used to fix the device behind the Siemens mirror holder. The stereoscope mirror holder is curved downwards to lower mirrors toward the participant’s eyes through the eye pieces of the head coil. The vertical position of the mirror holder can be adjusted using a slider that attaches to the clamp. Mirrors are glued to the lateral and medial mirror stands. The position of lateral mirrors can be finely adjusted using a small handle to facilitate fusion. Medial mirrors are fixed at 45°. ***B***, Prototype of the MRI stereoscope mounted onto a Siemens 64-channel head coil, ***C*** with close-up image showing stereoscope with attached mirrors in lateral and medial positions.

The MRI stereoscope (construction plans available at https://github.com/betinaip/MRIstereoscope.git) was made of two parts which are joined firmly together using nylon screws: the “mirror holder” held the stereoscope mirrors (Fig. 2*A*, “mirror holder”) and the “clamp” attached the mirror holder tightly to the 64-channel head coil by clamping onto the Siemens mirror holder. Both parts were machine cut from transparent acrylic glass (Fig. 2*B*). The clamp was further stabilized by an acrylic rod fit behind the rear-view mirror. Two nylon screws on each side held the mirror frame, which was shaped to allow closer positioning of the mirrors to each eye by curving downwards toward the eye and arching over the nasal piece of the head coil. Two mirrors on each side are attached using double sided tape after the stereoscope has been assembled. The mirrors delivered the monocular images: the medial and the lateral mirror (Fig. 2*C*). The medial mirrors were tilted at 45° relative to the monocular line of sight. The lateral mirrors could be finely adjusted using a small level on the side of the mounts, and were roughly parallel to the medial mirrors. The ability to adjust the lateral mirrors allowed participants to tweak the stereoscope until binocular fusion was achieved inside the scanner. The medial mirrors were fixed at an interpupillary distance (IPD) of 6.2 cm. A list of suggested materials for construction of the MRI stereoscope system is shown in Table 2.

**Table 2:** Suggested item list and representative costs for construction of MRI stereoscope

Item	Item description	Cost (USD)	#	Total (USD)
Machine workshop fee	MRI stereoscope mirror holder	$3700.00	1	$3700.00
Foam board for septum	Black Foamex PVC foam board thickness: 5.0 mm, 1100 × 590 mm	$30.00	1	$30.00
Blackout curtain	Black nylon, polyurethane-coated fabric, 1.5 m × 2.7 m × 0.12 mm thick	$60.00	1	$60.00
Siemens mirror holder	Siemens mirror holder 64	$20.00	1	$20.00
Front silvered head coil mirror	Plane front mirror surface, 160 × 120 × 2 mm	$210.00	1	$210.00
Front silvered stereoscope mirrors	Front surface mirror, 25 × 25 × 2 mm	$60.00	4	$240.00
Total estimated cost				$4260.00

Materials ordered for construction of stereoscope system, item description, estimated cost in United States dollars (USD). Final costs will vary depending on region and manufacturing choices. PVC = polyvinyl chloride, $ = U.S. dollar.

### Set-up

The stereoscope system was completed by adding the septum, which vertically split the visual space from the horizontal center of the head coil mirror to the horizontal center of the LCD screen. Blackout curtains were added to eliminate undesired illumination not coming directly from the LCD screen. The installation and dismantling could be achieved within 5 min. The participant was asked to settle into the head coil wearing acrylic goggles as a protective barrier between the participant’s eyes and the stereoscope mirrors. If the participant required visual correction in the scanner, the MRI safe prescription glasses were used instead of protective goggles. Additional head cushions were used to prop the participant closer to the mirrors if necessary. Once the participant was comfortably settled, the top part of the head coil, together with the MRI stereoscope, was clamped onto the bottom part.

### MRI stereoscope calibration

**Figure 3.**
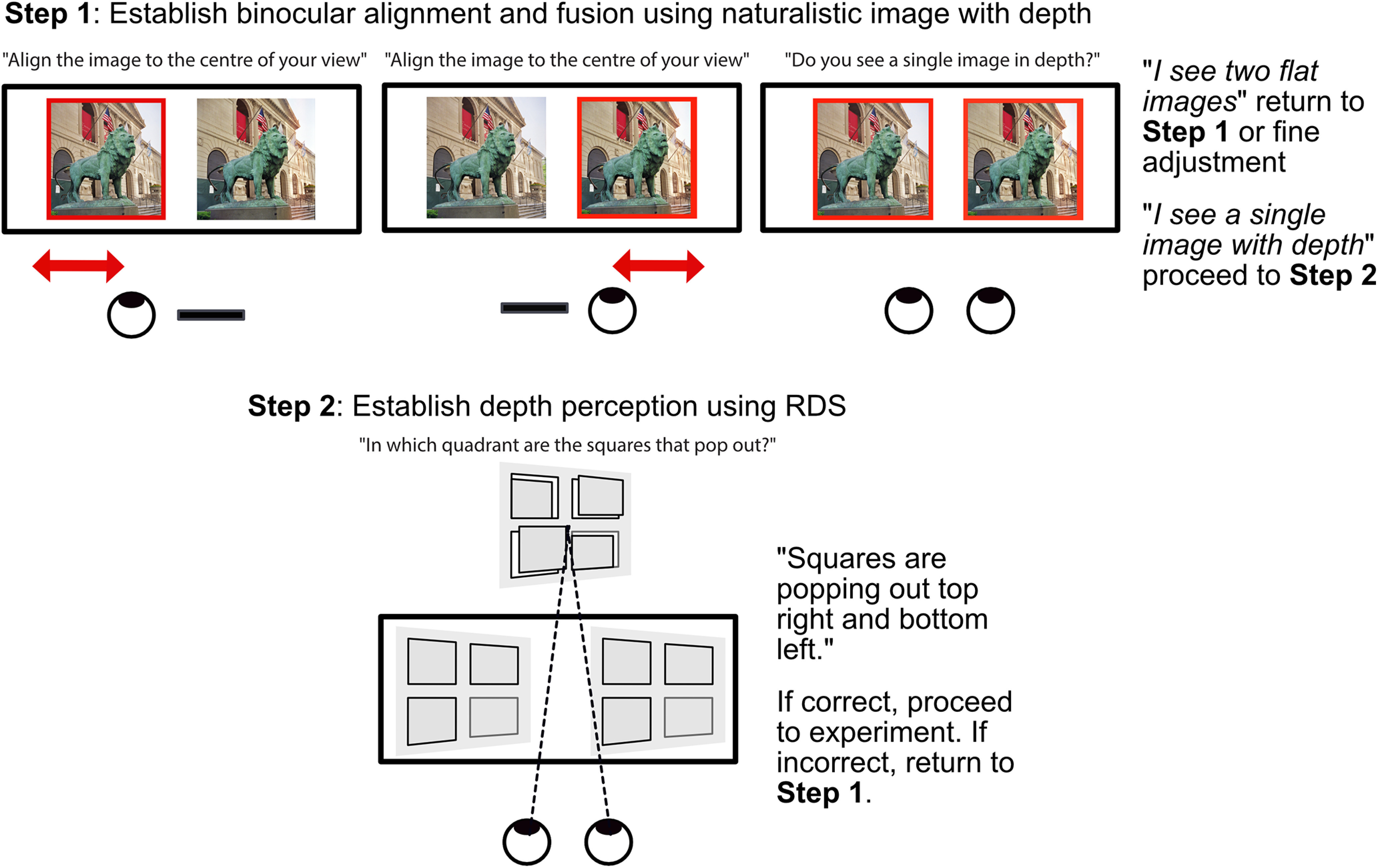
Diagram of two-step calibration procedure. Step 1 establishes alignment of visual axes and binocular single vision. Step 2 aims to evaluate the perception of depth in disparity defined random dot stereograms (RDS). If the participant incorrectly identifies the depth in step 2, step 1 is repeated to allow re-adjustment until step 2 allows correct report of binocular depth. The calibration procedure takes ∼5 min per participant.

Participants performed a brief two-step calibration procedure using the button box to achieve fusion in the scanner (Fig. 3). Step 1 of the calibration commenced with naturalistic images containing binocular disparity. Participants were instructed to view the display with each eye alone. They then used a button box to shift the horizontal position of left or right half of the image until it was aligned with the center of view. Upon opening both eyes, the two visual axes should be in alignment and a single fused image with clearly reportable stereoscopic depth perceived. If participants perceived a double image they were instructed to adjust the calibration until a single image was perceived. In step 2, a four-alternative forced choice task using four RDS squares rendered at a fixed binocular disparity (±0.15°) was used to evaluate the perception of stereoscopic depth in RDS. Participants were asked which two out of four squares “popped out.” After a series of three trials with verbally delivered correct answers, the main experiment commenced. Calibrations usually took around 5 min, and once achieved, binocular fusion was stable throughout the scan time and was verbally confirmed before each stimulus run. More extensive and frequent calibration procedures could be applied as necessary in populations with abnormal stereovision (see Discussion).

### Training requirements for users

The manipulation of the lateral mirrors is usually not required, as the image calibration procedure by the participant is sufficient to allow fusion. If necessary, manipulation of lateral mirrors should always be done by the experimenter and not the participant. This is because handling of the lateral mirrors requires fine adjustments. Poor adjustment of lateral mirrors can cause extreme vergence positions that are avoided by confirming fusion and comfort levels with the participant. Although no instances of visual discomfort occurred during the validation scans, it is generally recommended that the participants should be asked about their visual comfort level during the experiment and given opportunity to rest between scans in case they feel tired.

### Experimental validation of device

#### Participants

Data were extracted from a larger data set to study binocular disparity organization in the human visual cortex that used the MRI stereoscope. As reported here, six volunteers with normal or corrected-to-normal vision participated in an experiment designed to assess the representation of binocular disparity in the human visual cortex (age range 19–39 years, mean age 31 years, four females). All participants had normal visual acuity and stereoscopic depth perception (TNO test <60 arcsec, Frisby stereotest <40 arcsec). The study received ethical approval from the University of Oxford Central University Research Ethics Committee (R53110/RE002) and experimental procedures were consistent with the Declaration of Helsinki (2013 revision).

#### Visual stimulation protocol

**Figure 4.**
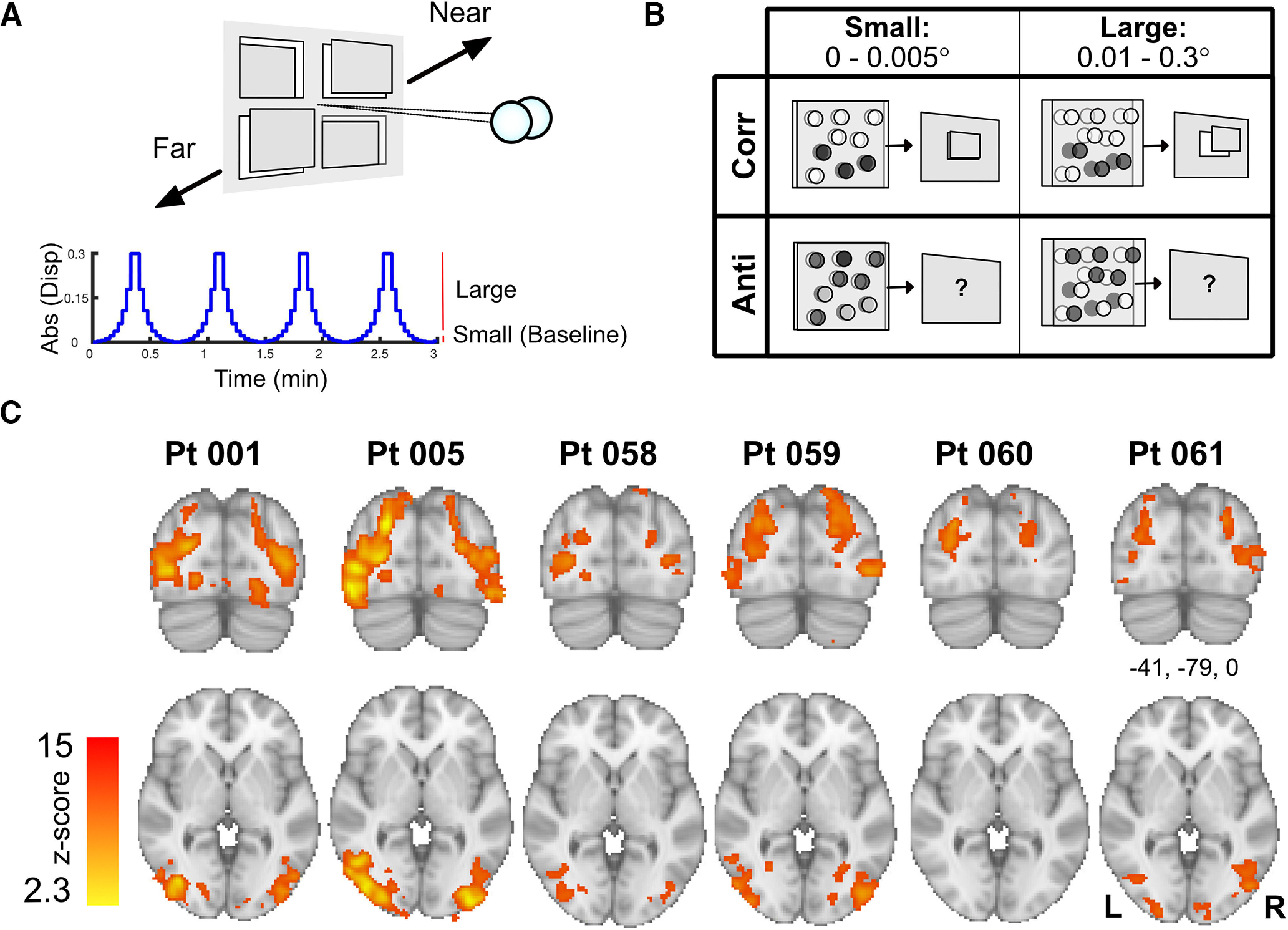
Proof-of-concept experiment shows strong and consistent activation in visual cortex when participants viewed large compared with small binocular disparities using the MRI stereoscope. ***A***, Binocular disparity stimuli were squares of dynamic random dot that moved far and near relative to a zero-disparity frame over time. ***B***, Comparison of correlated (“corr”) large binocular disparities (0.01–0.3°) to small disparities (0–0.005°) controlled for monocular and small binocular disparity cues while isolating regions sensitive to larger disparities. In a control experiment, participants viewed stimuli that were identical, except that the contrast of dots was inversed (“anti”) between left and right eye (i.e., white dots in right eye are black dots in left eye and vice versa). Anti-correlated stimuli provide binocular disparity that do not result in depth perception. ***C***, The contrast between large > small disparities in correlated stimuli evoked statistically significant activation in dorsal and ventral extrastriate regions of the visual cortex across all six participants. Activation maps were thresholded at Z > 3.1 and cluster-corrected at *p* < 0.05. Abs(Disp) = absolute disparity; Pt = participant.

Stimuli were dynamic random dot stimuli that varied in disparity magnitude over time with a sinusoidal profile (Fig. 4*A*). Each participant completed eight experimental runs of 6 min each. Four runs presented correlated disparity, and four runs anti-correlated disparity. The anti-correlated disparity condition presents disparity that does not generate depth perception. It controlled for stimulus properties (dot position, stimulus envelopes) by inverting the contrast of dots between eyes. Within each run, the number of trials with positive and negative disparities were matched. Stimuli were composed of 5000 dynamically rendered black and white dots (dot size = 0.05°, dot density = 38 dot/°, refresh rate = 30 Hz, 50% white and 50% black dots on any given frame) displayed on a binocularly fused area of 10.23° × 12.88°. Stimuli appeared as a field of random dots, where four rectangular regions (each 3.32° × 4.64°) moved near and far in depth over time. The disparity modulations were created by plotting RDS with disparity, which varied smoothly over time from −0.3° to +0.3°, crossing zero: 0°, ±0.002°, ±0.005°, ±0.011°, ±0.020°, ±0.036°, ±0.062°, ±0.106°, ±0.179°, ±0.300°. The appearance of disparity defined depth on a display screen may elicit vergence eye movements. To mitigate this possibility, the fixation dot (0.2° radius) was at zero disparity and was surrounded by a zero-disparity region (0.4° radius). RDS squares of equal disparity sign were always placed at diagonal positions to each other, such that vergence cues were balanced across the top and bottom, as well as left and right halves of the screen. For the purpose of the analysis, binocular disparity conditions were averaged across an equal number of “near” and “far” disparity presentations, and binned into small (0–0.005°), and large disparities (0.011–0.3°).

Participants performed a global contrast detection task to ensure even allocation of spatial attention across the whole screen and to keep them awake. Participants were asked to keep their eyes on the fixation dot and press a button on the button box whenever a global change in stimuli contrast was apparent. These were 72 pseudo-randomly timed events distributed across the run. Each of the target events was 1 s long and was composed of a 20% drop in contrast, introduced gradually to prevent eye blinks caused by sudden changes.

#### MRI acquisition

Data were acquired using a 3T Siemens Prisma scanner, with a 64-channel head and neck coil. Functional images were collected using a gradient echo EPI sequence (TR = 1355 ms, TE = 32.40 ms, flip angle = 70°, 72 slices, resolution = 2 mm isotropic) with parallel multiband acceleration (MB factor = 4; Moeller et al., 2010). T1-weighted anatomic images were acquired for registration and visualization purposes (MPRAGE, TR = 1900 ms, TE = 3.97 ms, TI = 904 ms, flip angle = 8°, slices = 192, resolution = 1 mm isotropic). In addition, B_0_ field maps were collected to estimate and correct distortions because of field inhomogeneity (TR = 482 ms, TE_1_ = 4.92 ms, TE_2_ = 7.38 ms, resolution = 2 mm isotropic).

#### MRI analysis

fMRI data analysis was performed using FEAT (FMRI Expert Analysis Tool) v6.00, part of the FSL software distribution (FMRIB’s Software Library; www.fmrib.ox.ac.uk/fsl). Data were preprocessed using motion correction MCFLIRT (Jenkinson et al., 2002), nonbrain tissue extraction (Smith, 2002), spatial smoothing using Gaussian kernel of FWHM = 5 mm, grand-mean intensity normalization and high pass temporal filtering (Gaussian-weighted least squares straight line fitting, main experiment = 100 s. Functional images were registered to the 1 mm isotropic T1-weighted structural image using boundary-based registration (BBR) in FLIRT (Jenkinson and Smith 2001; Jenkinson et al., 2002). The group activation map was derived using FLAME 1 + 2 (FMRIB’s Local Analysis of Mixed Effects). Z-scored statistical images of Gaussianized T-score and F-score statistical images were thresholded using clusters determined by Z > 3.1 and a (corrected) cluster significance threshold of *p* = 0.05 (Worsley, 2001).

## Results

### Seeing large binocular disparities through the MRI stereoscope activated brain regions sensitive to binocular depth

We have demonstrated the capacity of the MRI stereoscope system to display dichoptic information in the MRI scanner by collecting data from six participants during viewing of dynamic RDS rendered with binocular disparity. Four disparity-defined squares moved near and far over time (Fig. 4*A*), with the same changes in disparity magnitude over time. For the purpose of our validation, we averaged across near and far disparities and visual field locations and analyzed the data solely using the disparity magnitude. Small and large disparities generated binocular depth perception, but differed in the perceived depth magnitude (Fig. 4*B*). By comparing large binocular disparities (“large,” 0.01–0.3°) to a baseline of disparities near zero (“small,” 0–0.005°), we aimed to identify regions in the visual brain that are sensitive to larger binocular disparities while controlling for activation because of monocular cues and small binocular disparities. These precise binocular cues would only evoke responses in regions that are sensitive to an increase in disparity if the device accurately presented this information. No difference in cortical responses would be present if the device failed to provide accurate dichoptic information.

The comparison of large with small binocular disparities revealed strong bilateral activation in extra-striate visual cortex associated with depth perception across individual participants, including lateral occipital regions and intraparietal cortical regions. The results are displayed for each individual participant on the MNI-standard brain in coronal (top) and axial (bottom) orientation in Figure 4*C*.

### Anti-correlated stimuli that do not evoke depth perception also do not activate binocular depth sensitive visual areas

**Figure 5.**
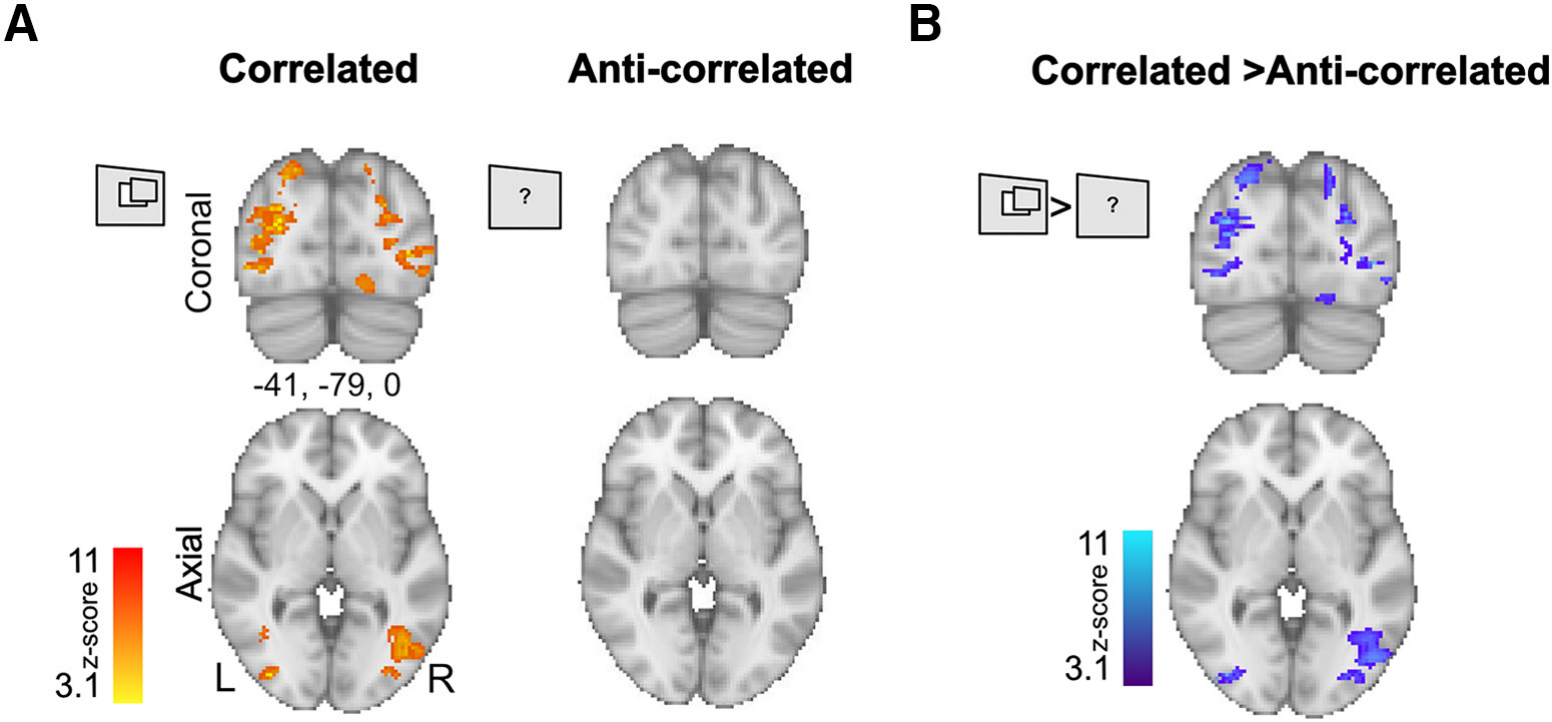
Control experiment shows no activation in cortical areas modulated by large binocular disparities when the same stimuli were binocularly anti-correlated. ***A***, Group activation maps show that correlated disparity (large > small) evoked large regions of activation across participants. In contrast, anti-correlated disparity stimuli (large > small), averaged across identical number of participants, same number of runs, and collected in the same experimental session, did not evoke any significant group responses. ***B***, Significant differences between correlated and anti-correlated disparity were shown across corresponding regions in the visual cortex (paired *t* test). All activation maps were thresholded at Z > 3.1 and cluster-corrected at *p* < 0.05.

Large binocular disparity evoked strong activation across intraparietal regions and lateral occipital cortex across participants (Fig. 5*A*). As a stringent control condition, participants also viewed binocularly anti-correlated RDS, which provide identical visual information but are known not to evoke any perception of depth (Cumming and Parker, 1997; Fig. 4*B*, see diagram). No significant group activation was present for anti-correlated visual stimuli (Fig. 5*A*), and there was significantly greater activity to correlated disparity when the two conditions were compared directly using a paired *t* test (Fig. 5*B*). The pattern of greater activity to correlated disparity reflects the same areas evident in the individual participants, namely the lateral occipital cortex and intraparietal regions.

## Discussion

Our MRI stereoscope system allowed for affordable, high-quality dichoptic display in the MRI-environment with zero cross talk, while duplicating the display properties of the projector screen in spatial and temporal resolution. The system thus meets essential criteria for researchers interested in binocular (3D) vision to investigate depth perception, interocular suppression, continuous flash suppression or binocular rivalry. It takes advantage of widely available commercial devices with minor modifications, and with most components constructed from low-cost materials. Results replicated known properties of binocular visual processing in the human brain. The system is safe and rapid to deploy, and available at a manufacturing cost far below that of commercially available dichoptic display systems, while providing excellent quality stereoscopic stimulation in the scanner. The viewing arrangements allow for standard spectacle correction lenses to be inserted within the optical path. Unlike an alternative low-cost solution using prism glasses, there is no distortion of the viewed image when the mirrors are perfectly aligned (Schurger, 2009). Even if the mirrors are not perfectly aligned, the distortions are relatively small.

### Proof-of-concept experiment

As a proof-of-concept, we analyzed fMRI data from six participants, who viewed RDS rendered in binocular disparity that changed in magnitude of disparity-defined depth over time. Results showed highly significant activation across multiple regions of the extrastriate visual cortex in all participants. The sensitivity of dorsal and ventral extrastriate areas to binocular disparity are well documented in the fMRI literature (Neri et al., 2004; Bridge and Parker 2007; Preston et al., 2008; Minini et al., 2010; Goncalves et al., 2015). Our results largely confirm these previous findings, supporting the view that binocular disparity is involves a network of areas that serve computation of higher-level visual information. These results provide clear evidence that the MRI stereoscope accurately presented dichoptic information in the MRI scanner.

### Potential for development

The system was developed to work with a 64-channel head and neck coil for a 3T-Prisma scanner, with an LCD screen positioned at the back of the scanner bore. There are obvious limitations in the size of the visual field. Because of the side-by-side stereo presentation, the dichoptic visual field is half the visual field of the normal display screen. Our setup achieved a maximum dichoptic visual field coverage of 10°. While the display screen size is essentially limited by the diameter of the scanner bore (in this case 60 cm), a larger visual field could be achieved by decreasing the distance between the participant’s eyes and the display screen or by optically extending the screen size. Greater visual field size will be achieved in wide-bore scanners with a bore dimension of 70 cm. The optical quality of the display image is true to the MRI compatible LCD screen. For experiments needing higher pixel resolution, ultra-high-definition MRI-safe displays, such as the 3840 × 2160-pixel resolution BOLD-screen 32 UHD by Cambridge Research Systems, can be used.

In cases where self-calibration is not successful, angular calibration of the lateral mirrors can be used to facilitate alignment and fusion of dichoptic images. However, angling the lateral mirrors away from parallel with the medial mirrors will introduce image skewing that induce vertical disparities and changes in horizontal disparity. Although relatively small, such distortions can be estimated mathematically (Held and Banks, 2008).

Because we are currently unable to perform binocular eye tracking in the MR scanner with this version of the stereoscope, we cannot guarantee that there is a complete absence of vergence eye movements. A further point is that if the subjects had succeeded in tracking the depth changes in one of the four RDS stimuli with vergence eye movements, this would result in a nearly constant stereoscopic depth for that RDS stimulus, while the other three would be varying over time. In this case, one would expect to see considerable asymmetry in the strength of fMRI activation across different positions in the visual field and between cortical hemispheres. These asymmetries were not seen, suggesting that participants did succeed in maintaining stable binocular fixation and steady vergence on the static fixation point, as requested by the experimental instructions. In our setup, eye tracking is made more challenging because of the limited view of the eye when the MRI stereoscope is mounted. The instrument also partially obscures the illumination from the infrared illuminator. This issue may be overcome by replacing the stereoscope mirrors with cold-mirrors (Brascamp and Naber, 2017), which reflect visible light and let IR light from the eye tracker illuminator pass through. Additional illuminators can also be placed inside the bore to increase the IR illumination, and therefore the ability to track the eye. While the system is fitted for a back-projection system, minor modifications will allow a front-projection arrangement.

The MRI stereoscope has been validated in a normally sighted adult population. It can be used in adult populations with visual impairments, provided participants are able to perceive the stimuli well enough for the calibration. The calibration procedure requires seeing the dichoptic images, and fine adjustment of the horizontal position of the monocular images. For participants with abnormal binocular vision, a more rigorous and frequent calibration procedure, such as used in the psychophysical laboratory (Ding et al., 2013), can be applied to ensure fusion. The current MRI stereoscope is not suited for pediatric populations because of the fixed inter-pupillary distance.

In conclusion, the MRI stereoscope is a cost-effective instrument that delivers precise, high-quality and comfortable dichoptic stimulation inside the MRI scanner. Because of the spatial separation of monocular images, there is zero cross talk. The system does not require additional polarizing goggles or prism lenses for stereo delivery, and is therefore more comfortable to use for participants, in particular those who needed MRI-safe glasses for visual correction. The display properties are true to that of the native display system, and the dichoptic experience is easily adapted when better LCD-monitors or projectors become available for the MRI scanning environment. The main limitations are the small field-of-view, and the lack of integrated eye tracking in the current set-up. The system will be attractive for scientists investigating binocular depth perception, binocular rivalry and continuous flash suppression, among other dichoptic psychophysical paradigms. Our MRI stereoscope system substantially improves the options for achieving high-quality dichoptic displays in the MRI scanner, and can lead to accelerated insights into the brain mechanisms of stereoscopic perception and consciousness. We provide open-source materials such that the MRI stereoscope can be used as a basis for future research and innovation in perceptual neuroscience.

## Synthesis

Reviewing Editor: Nicholas J. Priebe, University of Texas at Austin Decisions are customarily a result of the Reviewing Editor and the peer reviewers coming together and discussing their recommendations until a consensus is reached. When revisions are invited, a fact-based synthesis statement explaining their decision and outlining what is needed to prepare a revision will be listed below. The following reviewer(s) agreed to reveal their identity: Bas Rokers, Randolph Blake.

SYNTHESIS

Two reviewers evaluated the manuscript “MRI stereoscope: a miniature stereoscope for human neuroimaging” and both recognized the importance of disseminating the technical details of a mirror-based system for dichoptic stimulation in an MRI setting. There was consensus, both in the reviews and in the consultations that followed, that while the manuscript in its present form is a good start, there are technical issues that require clarification. Additionally, both reviewers commented that while this manuscript provides an important resource for dichoptic stimulation, this is not the first system to be used for this purpose, and thus the novelty in the manuscript should be tempered. The reviewers provide extensive comments which you should find useful in revising your manuscript, if you decide to resubmit it to eNeuro, or to another journal.

Reviewer #1:

Advances the Field:

Provides a previously unavailable description of an affordable mirror-based device for MRI-safe dichoptic stimulus presentation.

Comments:

The authors describe an MRI-compatible miniature stereoscope system that can be used for dichoptic presentation of visual stimuli. The main goal of the manuscript is to provide a detailed and replicable description of the system.

In addition, to validate the device, six participants were scanned while viewing dynamic random dot stereograms (DRDS) that varied in depth over time. According to the authors, the bilateral activation of lateral occipital cortex and intraparietal cortical regions confirm that the device evokes robust responses to binocular disparity validating the functioning of the device.

As the authors point out there is a significant barrier to conducting dichoptic MRI experiments because of cost, artifacts and potential miscalibration. The manuscript provides a viable option and discusses the pros and cons of the solution.

Major points

I have a few major points that I hope the authors can address.

The authors provide a thorough overview of the most popular devices. It would be good to point out limitations that all of these systems have in common. While they can present some depth cues, such as binocular disparity, they are unable to reproduce others that are possible in the psychophysics laboratory: No depth from focus, no motion parallax.

Adjustment of mirrors requires some expertise, to avoid extreme vergence position for example. What looks good and is comfortable at the beginning of the experiment, may not after an hour of scanning despite a calibration test. It should be mentioned that the presented solution might not be suitable for naive participants without some training or clinical populations with visual impairments.

Another potential issue with the presented solution is that it relies on a fixed IPD. An IPD mismatch will induce distortions and may induce discomfort (Fulvio, Ji & Rokers, 2021). Moreover, if the lateral mirrors are not exactly at 45 deg (the medial mirrors are fixed) they induce toe-in distortions of the image. Those may be small for the FOV achievable with the system, but would be good to mention and if possible quantify.

The description of the experimental procedure would benefit from more detail. Specifically:

Calibration procedure (page 8). Did the procedure use the DRDS stimuli used during the experimental procedure as well? If so, which was the level of disparity (small or large disparity)? How many trials did you perform and what criterion was used to decide when to stop the calibration procedure and start the experiment?

Visual stimulation protocol: How many repetitions of near and far positions were presented in each run? How many runs did you collect for each subject and how long was each run?

Global attention task: Please provide more detail on the global contrast task participants performed in the scanner: how was the contrast changed during the experiment? Moreover, the stimulus configuration might have induced eye movements. It would be helpful to make explicit how such eye movements might have affected the results.

Minor comments

Lines 44-45: “However, spatial separation typically comes at the expense of visual field coverage, as each eye's image occupies one half of the visual display.”

Should mention that issues of appropriate alignment of the two eye's images arise.

Table 1: Item 5, mention that because of the MR-safe requirements these solutions often lead to the use of fiber optic cables, which can lead to light spray and dead pixels.

Line 62: The mirror stereoscopes -> Mirror stereoscopes

Line 65-66: “, but are thought to be incompatible with the MRI-environment because stereoscopes usually contain ferromagnetic materials and are relatively large in size"

I do not believe this is generally held. How about: stereoscopes have been used, but we did not find methodological descriptions with sufficient detail to replicate these instruments

Line 80: effectively halving a LCD display screen - Drop LCD. Issue occurs regardless of display technology.

Line 88-89: This is weird: “Because the images were viewed through the rear-view mirror in a supine position, the left half of the LCD screen image was seen by the right eye, the right half by the left eye.”

This is only because you compare to someone looking at the screen directly without the mirrors, which seems not relevant. How about: seen through the mirrors the left half of the screen is viewed by the left eye only, and the right half bit the right eye only.

Line 180: AS reported -> As reported

Line 188: Would be helpful to have more information about the visual stimuli, such as dot density. As the percepts moved in depth, how does that work, do they just flip between near/far or does the stimulus go through intermediate positions? (Figure 3c speaks to it, but would help to clarify).

The anticorrelated control is nice. Would be helpful to point out that this directly addresses a concern of shifting stimulus envelopes in the monocular half images.

Line 287-288: I do not think that is technically right if the mirrors are not perfectly aligned. Instead say much less distortion.

Line 296: “binocular disparity is not exclusively processed in a single area” that seems very strawmanny. This is not true for pretty much any visual stimulus feature.

Reviewer #2:

Advances the Field:

Technical description of another means for achieving dichoptic stimulation, one with limitations. Data used to validate the device are replications of several existing aspects of disparity processing in the human brain.

Comments:

This is nicely written description of the lab's miniature stereoscope that they used successfully to obtain some basic findings on disparity processing by the human brain. Those results largely replicate earlier published brain imaging results, but they do serve as confirmation that their particular fabrication for achieving dichoptic stimulation does the job it was designed to do. I would, however, respectfully point that this is certainly not the first successful attempt to deploy a custom-built stereoscope for brain imaging work - it is something of an overstatement to tout their accomplishment as 'novel' as they do in their significance statement. To their credit, they do go to some length to survey strengths and weaknesses of various techniques used to achieve dichoptic stimulation in a scanning environment (with plenty of references), and they are up-front in acknowledging the principal weaknesses with their mirror-based device, i.e., its limited field of view and inability, to date, to interface with eye-tracking.

It is not uncommon for labs to publish papers describing and validating technical details of instruments/devices used regularly in their work - those descriptions are seen in journals such as J. Neurosci. Methods and Behav. Res. Methods. It would not be unprecedented, then, for this submission to appear in a refereed journal, and I believe some in this field of study would appreciate the opportunity to read about this instantiation of a mirror stereoscope in the scanner. It is gratifying to read that the authors intend to share information about fabrication of their particular device.
